# *Toxoplasma gondii* antibodies in tropical seabirds from the Rocas Atoll Biological Reserve, Brazil

**DOI:** 10.1590/S1984-29612024052

**Published:** 2024-09-23

**Authors:** Daniela Bueno Mariani, Solange Maria Gennari, Herbert Sousa Soares, Renata Hurtado, Veridiana Caso Galizia, Maurizélia de Brito Silva, Eduardo Cavalcante de Macedo, Ricardo Augusto Dias, Jean Carlos Ramos Silva

**Affiliations:** 1 Programa de Pós-graduação em Medicina Veterinária, Universidade Federal Rural de Pernambuco – UFRPE, Recife, PE, Brasil; 2 Departamento de Medicina Veterinária Preventiva e Saúde Animal, Faculdade de Medicina Veterinária e Zootecnia, Universidade de São Paulo – USP, São Paulo, SP, Brasil; 3 Programa de Pós-graduação em Saúde Única, Faculdade de Medicina Veterinária, Universidade Santo Amaro – UNISA, São Paulo, SP, Brasil; 4 Southern African Foundation for the Conservation of Coastal Birds – SANCCOB, Cape Town, South Africa; 5 Veterinária autônoma, São Paulo, SP, Brasil; 6 Instituto Chico Mendes de Conservação da Biodiversidade – ICMBio, Ministério do Meio Ambiente – MMA, Natal, RN, Brasil; 7 Instituto Brasileiro para Medicina da Conservação – Tríade, Recife, PE, Brasil

**Keywords:** Marine environment, seabirds, toxoplasmosis, Ambiente marinho, aves marinhas, toxoplasmose

## Abstract

*Toxoplasma gondii* is a coccidian that infects almost all warm-blooded animals, including birds. Rocas Atoll Biological Reserve, located in the northeast of Brazil, is the only atoll in the South Atlantic, and home to the largest population of seabirds in the western Atlantic. In this study the occurrence of *T. gondii* antibodies in seabirds from Rocas Atoll were determined. Birds were manually captured, blood samples were taken, and antibodies detected by the modified agglutination test (>5). In total, 267 birds of seven species belonging to three families (Sternidae, Fregatidae and Sulidae) and two orders (Charadriiformes and Suliformes) were sampled. Out of the 267 samples, 20 (7.3%) were seropositive: nine out of 48 brown noddies (*Anous stolidus*), one out of 26 black noddies (*Anous minutus*), three out of 20 magnificent frigatebirds (*Fregata magnificens*), five out of 95 sooty terns (*Onychoprion fuscatus*) and two out of 20 red-footed boobies (*Sula sula*). None of the 33 masked boobies (*Sula dactylatra*) and the 25 brown boobies (*Sula leucogaster*) were seropositive. The antibody titers were 5 (n=6), 10 (n=4), 20 (n=3), 40 (n=6) and 160 (n=1). Due to the uniqueness of this environment, monitoring the seabirds is suggested to maintaining this Conservation Unit.

## Resumo

*Toxoplasma gondii* é um coccídio que infecta quase todos os animais de sangue quente, incluindo aves. A Reserva Biológica do Atol das Rocas, localizada no Nordeste do Brasil, é o único atol do Atlântico Sul que abriga a maior população de aves marinhas tropicais do oeste do Oceano Atlântico. O objetivo deste estudo foi verificar a ocorrência de anticorpos anti-*T. gondii* em aves marinhas do Atol das Rocas que foram capturadas manualmente. Amostras de sangue foram obtidas e anticorpos avaliados pelo teste de aglutinação modificado (>5). Foram amostradas 267 aves de sete espécies, pertencentes a três famílias (Sternidae, Fregatidae e Sulidae) e duas ordens (Charadriiformes e Suliformes). Das 267 aves, 20 (7,3%) foram soropositivas sendo: nove das 48 viuvinhas-marrons (*Anous stolidus*), uma das 26 viuvinhas-negras (*Anous minutus*), três das 20 fragatas (*Fregata magnificens*), cinco dos 95 trinta-réis-das-rocas (*Onychoprion fuscatus*) e dois dos 20 atobás-de-pés-vermelhos (*Sula sula*). Nenhum dos 33 atobás grandes (*Sula dactylatra*) e dos 25 atobás-pardos (*Sula leucogaster)* estavam soropositivos. Os títulos de anticorpos foram 5 (n=6), 10 (n=4), 20 (n=3), 40 (n=6) e 160 (n=1). Devido à singularidade deste ambiente, o monitoramento dessas aves marinhas é sugerido para promover uma abordagem de Saúde Única nesta Unidade de Conservação.

## Introduction

*Toxoplasma gondii* infections are prevalent in humans, domestic animals, and terrestrial and aquatic wildlife. The parasite is transmitted through ingestion of undercooked infected meat or consumption of food and water contaminated with oocysts present in the feces of infected cats, the definitive hosts (Dubey, 2010). The oocysts can remain viable in the environment for months under natural conditions (Dubey, 2010).

*Toxoplasma gondii* is recognized as an important pathogen in coastal marine mammals (reviewed by Shapiro et al., 2019). Oocysts from cat feces are believed to be washed into seawater and serve as a source of infection via transport hosts (Cole et al., 2000; Lindsay et al., 2004; Shapiro et al., 2019).

Only 3.2% of a total of 9,970 species of birds known in the world are adapted for life in a marine environment (Peterson, 2003). Among other biological characteristics, seabirds usually have a long-life cycle, with parental care, monogamous behavior, late sexual maturity and few offspring during each reproductive season. They can be considered residents, faithful to a given region, or may be migratory with the ability to move between different regions (Schreider & Burger, 2002). In Brazil, a variety of seabird species can be found, thus demonstrating the importance of this country in relation to conservation of sea and coastal birds worldwide (Pacheco et al., 2021).

**Figure 1:**
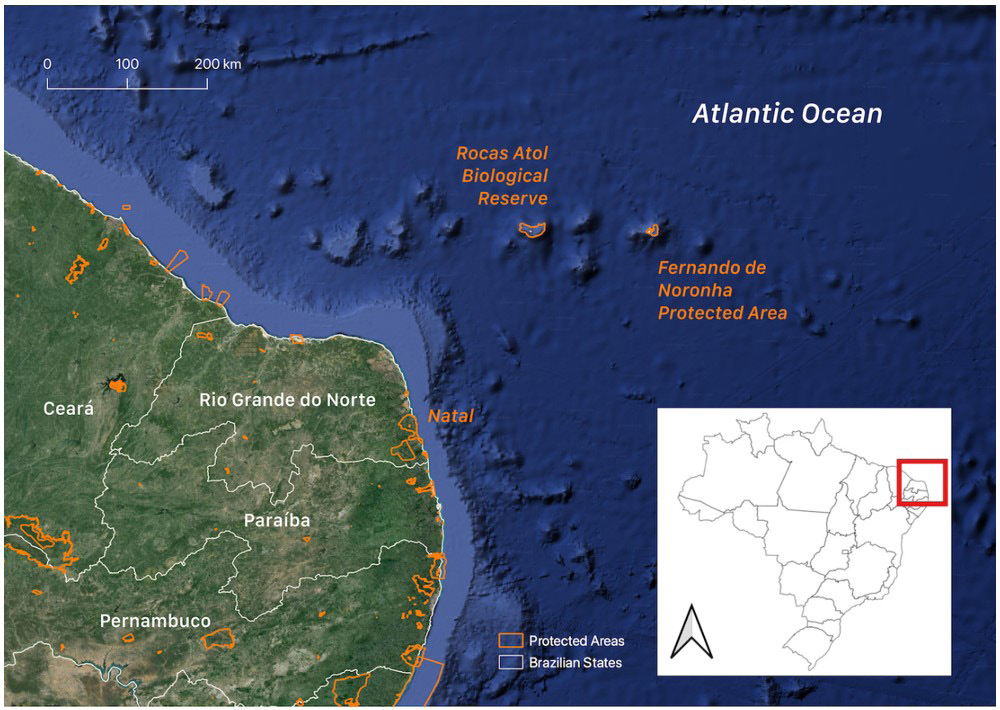
Map showing the location of the Rocas Atoll Biological Reserve, Brazil.

The Rocas Atoll Biological Reserve (3°51'42.0”S 33°47'21.6”W) is located 260 km northeast of the city of Natal, capital of the state of Rio Grande do Norte, and 145 km west of the archipelago of Fernando de Noronha, state of Pernambuco (Figure 1). The atoll is known for having the largest marine bird colony of the South Atlantic, composed of endemic birds, migrants and sporadic visitors that use the atoll for rest and food. It is already possible to catalog more than 143,000 birds of the five most abundant species that nest on the atoll (Schulz-Neto, 1998, 2004).

The objective of this study was to determine the occurrence of anti-*T. gondii* antibodies in serum samples from seabirds from Rocas Atoll.

## Material and Methods

Sample collection was carried out on the two islets that form Rocas Atoll (03º50’S and 33º49’W), i.e. the islet of Farol (227 samples) and Cemitério (40 samples), in three expeditions, from June to November 2017.

Seabirds were caught manually during periods of lower solar incidence to minimize stress on the colonies. Each bird was restrained, tagged with a metal ring and clinically examined. Blood was collected from the ulnar, medial metatarsal or jugular vein, according to the size of the bird. All the birds caught were adults.

Serum from these blood samples was tested for the presence of antibodies to *T. gondii* using the modified agglutination test (MAT), as described by Dubey & Desmonts (1987). The samples were firstly screened at 1:5 dilution and positive samples were then diluted two-fold. Positive and negative control serum from chicken was used in each test sera with titers ≥ 5 were considered positive (Dubey et al., 2003).

## Results and Discussion

The MAT used in the present study is considered specific for *T. gondii* infection and has been used for serological surveys among both mammals and birds. The efficacy of diagnosis of *T. gondii* antibodies in naturally infected birds (chickens) was evaluated by Dubey et al. (2016) and the dilution of 1:5 was considered to be the cutoff.

In total, 267 birds of seven species belonging to three families were sampled: Sternidae (*Anous minutus, A. stolidus* and *Onychoprion fuscatus*), Fregatidae (*Fregata magnificens*) and Sulidae (*Sula dactylatra, S. leucogaster* and *S. sula*).

Antibodies to *T. gondii* were found in 20 (7.5%) of the 267 birds, with titers that ranged from 5 to 160 (Table 1). Positive birds were found on both islets: 19 (8.4%) of the 227 samples from Farol and one (4.0%) of the 25 samples from Cemitério Islet.

**Table 1:** Occurrence of anti-*Toxoplasma gondii* antibodies and antibody titers in different species of seabirds on Rocas Atoll, Brazil.

**Species**	**Common Name**	**Number Tested**	**Number Positive (%)**	**Antibody Titer (No.)**
*Anous minutus* ^1^	Black Noddy	26	1 (5)	5 (1)
*Anous stolidus*^1^	Brown Noddy	48	9 (18.7)	5 (2); 10 (1); 20 (2); 40 (4)
*Fregata magnificens*^2^	Magnificent Frigatebird	20	3 (15)	10 (1); 20 (1); 40 (1)
*Onychoprion fuscatus* ^1^	Sooty Tern	95	5 (5.9)	5 (2); 10 (1); 40 (1); 160 (1)
*Sula dactylatra*^3^	Masked Booby	33	0 (0.0)	*
*Sula leucogaster* ^3^	Brown Booby	25	0 (0.0)	*
*Sula sula* ^3^	Red-footed Booby	20	2 (10)	5 (1); 10 (1)
**TOTAL**		**267**	**20 (7.5)**	

1 Order Charadriiformes, Family Sternidae;

2 Order Suliformes, Family Fregatidae;

3 Order Suliformes, Family Sulidae.

The highest occurrence was observed in *A. stolidus*, for which 9 of the 48 samples were positive to *T. gondii*. On the other hand, all the 33 samples from *S. dactylatra* and the 25 from *S. leucogaster* were seronegative. In another survey in the Abrolhos archipelago, also located in the northeast region of Brazil, in the state of Bahia, *S. dactylatra* and *S. leucogaster* were found to present occurrence of *T. gondii* antibodies of 34.8% (8/23) and 47.4% (9/19), respectively, using the same diagnostic method and cutoff (Gennari et al., 2016). However, it is worth remembering that the birds of these two species in which antibodies were not found are residents of the Rocas atoll, and that these individuals nest and live exclusively on these islets (Schulz-Neto, 1998, 2004).

Sato et al. (2024), using material from carcass of seabirds found along the coast of Santa Catarina, south Brazil, detected *T. gondii* DNA in tissues from seven of the 47 (14.8%) seabirds and confirming the presence of the *T. gondii* in two out of six species (*Larus dominicanus* and *Puffinus puffinus*) examined. In the present study all *S. leucogaster* birds examined were seronegative, and, in the study with seabirds from Santa Catarina coast, *T. gondii* DNA was not detected in the tissue of the six *S. leugocaster* examined.

In the present study, 10 of the 20 positive birds presented low MAT antibody titers of 5 (6 birds) and 10 (4 birds). Gennari et al. (2016) also found low MAT titers (5 and 10) in 23 of the 24 seabirds examined. Those authors recommended that serological evaluation and isolation of viable *T. gondii* antibodies from seabirds should be performed, given that such information regarding avian species is only well known for chickens (Dubey et al., 2021).

Other studies on islands without the presence of cats have found seropositive birds (Deem et al., 2010). It is assumed that since birds can fly, they can arrive from other regions already bearing infection. This is the case for frigatebirds and red-footed boobies, which come from the Fernando de Noronha, only 145 km away from Rocas atoll, where cats are present and *T. gondii* strains with different virulence were already obtained from mammals and birds (Almeida et al., 2000; Dubey et al., 2010, Lima et al., 2019). These seabirds may have become infected through ingestion of food contaminated with oocysts shed by cats or through ingestion of tissue cysts from infected animals.

Normally, seabirds feed exclusively on fish and squid. However, Lindsay et al. (2003) and Lindsay & Dubey (2009) observed that *T. gondii* oocysts can also sporulate and remain infectious for up to 24 months in seawater at 4 °C.

It has also been found that oocysts can be accumulated in filtering bivalve mollusks (Lindsay et al., 2004) and Massie et al. (2010) experimentally exposed Pacific sardines (*Sardinops sagax*) and anchovies (*Engraulis mordax*) to *T. gondii* oocysts and found that oocysts retained infectivity inside the fish’s alimentary canals. These mollusks and fish form a food source for seabirds and mammals and may be the reason why seabirds can become infected in island environments without the presence of cats.

In this study, none of the seabirds sampled showed clinical signs, despite a description of an acute fatal toxoplasmosis in a *S. sula* in Hawaii, USA (Work et al., 2000).

The birds sampled in this study can be divided into two groups: those resident on Rocas atoll: *A. minutus*, *A. stolidus*, *O. fuscatus*, *S. dactylatra* and *S. leucogaster*; and those from Fernando de Noronha archipelago that visit the atoll: *S. sula* and *F. magnificens* (Schulz-Neto, 2004). Rocas Atoll does not have populations of felids, just like the Abrolhos archipelago, where seabirds were also examined (Gennari et al., 2016). However, Fernando de Noronha has a cat population and *T. gondii* antibodies have already been reported in humans and in both domestic and wild animals (Costa et al., 2012; Carvalho et al., 2021).

It is important to emphasize the variety of migratory seabirds in the Rocas Atoll Biological Reserve (Schulz-Neto, 1998, 2004). Many of these species were not sampled in the present study but might be agents for dissemination of various pathogens. We are not aware of any reports from any previous serological studies on the occurrence of anti-*T. gondii* antibodies in samples from *Anous minutus, Anous stolidus, Fregata magnificens, Onychoprion fuscatus* or *Sula sula.*

Due to the uniqueness of this island environment, monitoring of these seabirds is suggested to promoting One Health approach to maintaining this Conservation Unit.
